# O‐glycosylation in viruses: A sweet tango

**DOI:** 10.1002/mlf2.12105

**Published:** 2024-03-25

**Authors:** Annan Ming, Jianxin Zhao, Yihan Liu, Yibo Wang, Xiaohui Wang, Jing Li, Leiliang Zhang

**Affiliations:** ^1^ Shandong Provincial Hospital Affiliated to Shandong First Medical University Jinan China; ^2^ Medical Science and Technology Innovation Center Shandong First Medical University & Shandong Academy of Medical Sciences Jinan China; ^3^ Beijing Key Laboratory of DNA Damage Response and College of Life Sciences Capital Normal University Beijing China; ^4^ Laboratory of Chemical Biology Changchun Institute of Applied Chemistry, Chinese Academy of Sciences Changchun China; ^5^ School of Applied Chemistry and Engineering University of Science and Technology of China Hefei China; ^6^ Beijing National Laboratory for Molecular Sciences Beijing China

**Keywords:** immune response, O‐GalNAc, O‐GlcNAc, O‐glycosylation, virus

## Abstract

O‐glycosylation is an ancient yet underappreciated protein posttranslational modification, on which many bacteria and viruses heavily rely to perform critical biological functions involved in numerous infectious diseases or even cancer. But due to the innate complexity of O‐glycosylation, research techniques have been limited to study its exact role in viral attachment and entry, assembly and exit, spreading in the host cells, and the innate and adaptive immunity of the host. Recently, the advent of many newly developed methodologies (e.g., mass spectrometry, chemical biology tools, and molecular dynamics simulations) has renewed and rekindled the interest in viral‐related O‐glycosylation in both viral proteins and host cells, which is further fueled by the COVID‐19 pandemic. In this review, we summarize recent advances in viral‐related O‐glycosylation, with a particular emphasis on the mucin‐type O‐linked α‐N‐acetylgalactosamine (O‐GalNAc) on viral proteins and the intracellular O‐linked β‐N‐acetylglucosamine (O‐GlcNAc) modifications on host proteins. We hope to provide valuable insights into the development of antiviral reagents or vaccines for better prevention or treatment of infectious diseases.

## INTRODUCTION

Viruses are intracellular pathogens that hijack the host cellular machinery to modify their own proteins for viral replication and release. They have been a major global public health concern, causing infectious diseases or even cancer. To develop effective antiviral drugs or vaccines, it is necessary for researchers to explore the mechanisms underlying virus activity. Among many regulatory pathways, O‐glycosylation stands out as a frequently observed but overlooked protein posttranslational modification (PTM). Glycans can be found in all living organisms, where they decorate cellular proteins, lipids, or even RNAs^1^. Glycosylation can be categorized into two types: N‐ and O‐glycosylation^2^. N‐glycosylation is installed onto the side chains of the asparagine (Asn, N) residue, while O‐glycosylation occurs on serine (Ser), threonine (Thr), or sometimes tyrosine (Tyr) residues^3^. Traditionally, the function of glycosylation in various biological contexts has been understudied, probably due to lack of appropriate tools, but modern matrix‐assisted laser desorption/ionization mass spectrometry (MALDI‐MS), chemical biology tools, and molecular dynamics (MD) simulations have revolutionized the field and becomes imperative in glycosylation studies.

Functioning at the interface of the host and the pathogen, glycans are vital for pathogen infectivity, viral particle formation, viral protein folding and secretion, and immune recognition. Compared to N‐glycans, O‐glycans in virology are even more neglected, probably due to the outdated notion that N‐glycans have a more significant impact on viral glycoproteins (GPs)^3^. Indeed, O‐glycans are far more complex than N‐glycans. First, the N‐glycan has a consensus motif of N‐X‐S/T (X stands for any amino acid except proline) in both eukaryotes and bacteria. But the O‐glycan does not have a consensus motif, which makes the site‐specific study of O‐glycans impossible without the modern mass spectrometry (MS) instruments. Second, while N‐glycans can be subtyped into different subtypes, including high‐mannose, hybrid, and complex types^4^, O‐glycans can be classified into eight types: O‐mannose, O‐fucose, O‐glucose, O‐galactose, O‐linked β‐N‐acetylglucosamine (O‐GlcNAc) in the endoplasmic reticulum (ER), O‐xylose (proteoglycans), O‐linked α‐N‐acetylglucosamine (O‐GalNAc, also named the mucin‐type O‐glycan), and the intracellular O‐GlcNAcylation.

All these O‐glycosylation events are mediated by distinct glycosyltransferases: protein O‐mannosyltransferase (POMT) for O‐mannose, protein O‐fucosyltransferase (POFUT) for O‐fucose, protein O‐glucosyltransferase (POGLUT) for O‐glucose, collagen β(1‐O) galactosyltransferase (Glt25d) for O‐galactose, epidermal growth factor domain‐specific O‐GlcNAc transferase (EOGT) for O‐GlcNAc, xylosyltransferase (XT) for O‐xylose, GalNAc‐transferase (GalNAc‐T or GALNT) for O‐GalNAc, and O‐GlcNAc transferase (OGT) for intracellular O‐GlcNAcylation in the cytoplasm, nucleus, and mitochondria^2^. Of these, the mucin‐type O‐GalNAc (hereafter referred to as O‐glycan) and the intracellular O‐GlcNAcylation are the focus of this review.

There are 20 GALNT enzymes for O‐GalNAc, and among them 15 enzymes have been shown to utilize the UDP‐GalNAc as the donor group and transfer GalNAc to the S/T/Y residues of the target proteins^5^. These enzymes reside in the Golgi and can glycosylate any protein in the secretory pathway^2^. They catalyze the first step of O‐GalNAc, and then the glycan chain is elongated and branched to form heterogeneous core structures. The chains are terminated by sialic acids and fucose residues, thus completing O‐glycan biosynthesis^5^.

In stark contrast, intracellular O‐GlcNAcylation utilizes only one writer, OGT, and only one eraser, O‐GlcNAcase (OGA). OGT resides in the cytoplasm, the nucleus, the mitochondria^6^, ^7^, and as recently demonstrated, the lysosome^8^. OGT transfers a single GlcNAc moiety from the UDP‐GlcNAc donor to the S/T residues of substrate proteins and modifies the stability, localization, and protein–protein interaction of the substrates. O‐GlcNAc is a monosaccharide and cannot be elongated. By integrating the metabolism of glucose, amino acid, glutamine, nucleotide, and lipid into the hexosamine biosynthesis pathway (HBP), the reversible and dynamic O‐GlcNAcylation functions as a rheostat to adjust cellular signaling pathways in response to environmental stress^6^, ^7^.

O‐glycosylation has always posed a formidable challenge to researchers, albeit it is fundamental to many biological pathways. First, glycan is often heterogeneous; thus, it is notoriously hard to synthesize at a homogenous level for in vitro studies. Second, glycosylation occurs at substoichiometry levels, causing the modified peptides to be easily masked in MS analysis. In this regard, the recent development of higher‐energy collisional dissociation (HCD), stepped collision energy/higher‐energy collisional dissociation (sceHCD), electron‐transfer dissociation (ETD), and electron‐transfer/higher‐energy collisional dissociation (EThcD) MS instrumentation has facilitated the site‐specific identification of glycosites. In the meantime, ample chemical biology tools have also been developed for glycobiology. Third, unlike DNA synthesis, which can be finished by PCR and ready‐made templates, there is no such streamlined process for glycan synthesis. Indeed, expressed protein ligation (EPL) and native chemical ligation (NPL) have enabled the synthesis of many O‐glycosylated proteins, such as erythropoietin (Epo) in erythropoiesis^9^ and α‐synuclein in Parkinson's disease^10^, but still there are lots of O‐glycosylated protein synthesis left to be desired. In this respect, MD simulation together with AlphaFold has greatly enabled understanding of the structural role of glycans, at least at an in silico level. Finally and particularly in viral studies, recombinantly expressed viral proteins might display distinct glycan profiles from their counterparts in the native viruses^3^, suggesting that the viral protein structure and viral context is at play here. Besides, the glyco signature might differ significantly when different cell lines are used, thus complicating vaccine development^11^.

Glycobiology has come a long way but surely has an even more arduous journey ahead. In fact, the 2022 Nobel laureate Dr. Carolyn Bertozzi not only is a pioneer in developing innovative chemical tools to study glycans but also contributed significantly to seminal studies of sialic‐acid‐binding immunoglobulin‐like lectins (Siglecs) in tumorigenesis^12^. For this review, which is structured according to the different types of nucleic acids in viruses, we will concentrate on representative viruses with O‐glycosylation and delineate recent findings on O‐GalNAc on viral proteins and O‐GlcNAc on host proteins in various viruses, covering identification of glycosylation sites, their biological functions, and their potential applications in therapy. We envision that as we dive deeper into the glycosylation field with more advanced techniques, more viral secrets will be revealed, providing a unique sweet perspective to tackle infectious diseases in the future.

## THE ROLES OF O‐GLYCOSYLATION IN DNA VIRUSES

### Hepatitis B virus (HBV)

Chronic infection of HBV has posed a huge health burden worldwide, contributing to many liver diseases and even hepatocellular carcinoma (HCC). HBV is a circular dsDNA virus, possessing four overlapping reading frames responsible for encoding surface protein (HBsAg), core antigen (HBcAg), envelope antigen (HBeAg), the viral polymerase, and the transcriptional transactivator X protein (HBx)^13^.

**Figure 1:**
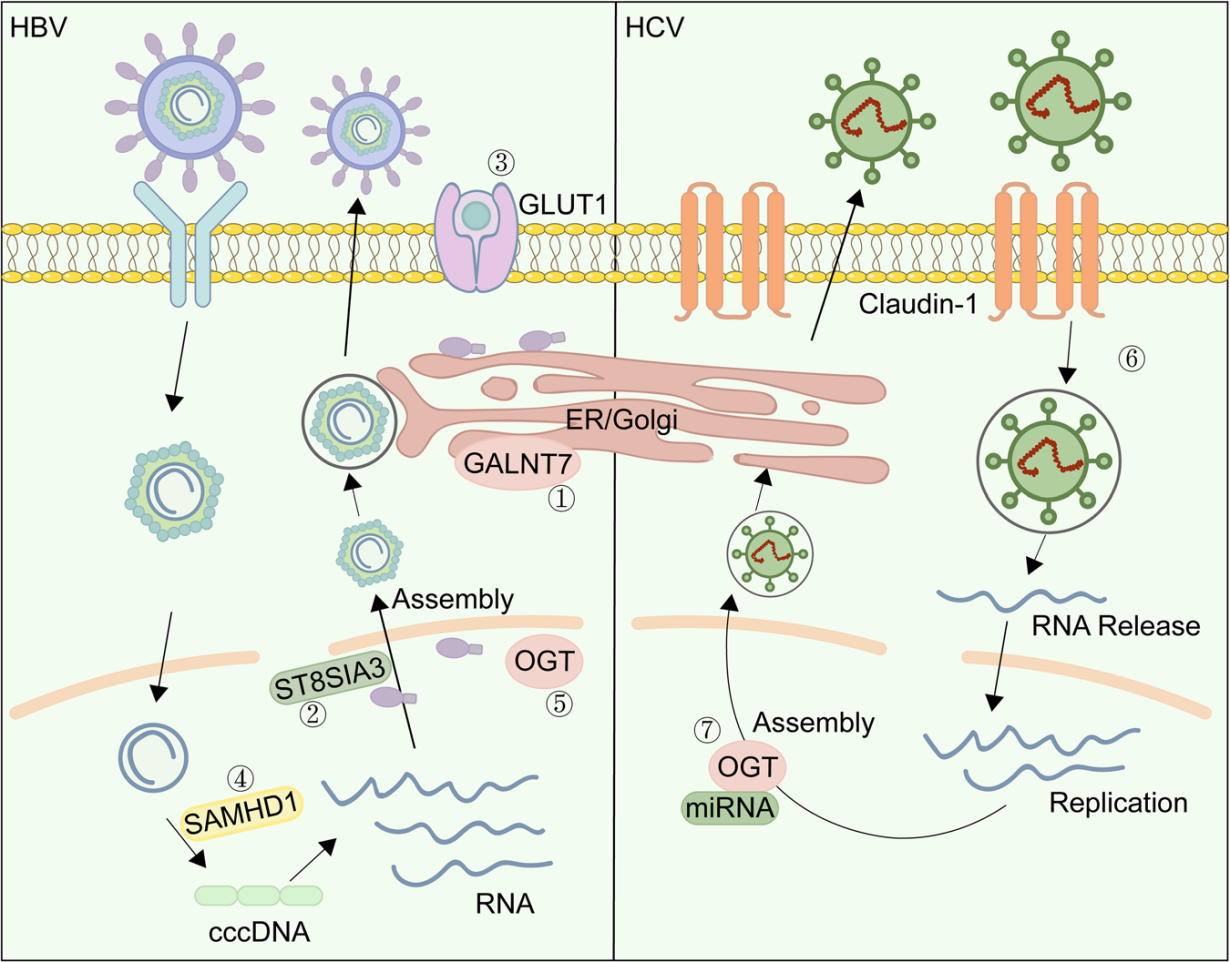
Schematic representation of O‐glycosylation in HBV/HCV life cycle. (1) GALNT7 transfers O‐GalNAc to viral protein to initiate O glycosylation. (2) ST8SIA3 is required for the secretion of HBsAg and HBV DNA. (3) Upon HBV infection, enhanced GLUT1 expression increases glucose uptake and protein O‐GlcNAcylation to inhibit viral replication. (4) O‐GlcNAcylation stabilizes SAMHD1, promotes its tetramer formation, and upregulates its dNTPase activity to limit viral replication. (5) OGT is essential for regulating both HBsAg and HBV DNA levels. (6) O‐GlcNAc modification on claudin‐1 is pivotal for HCV entry. (7) Two miRNAs modulate the posttranscriptional control of OGT to influence HCV assembly, release, and infectivity. GLUT1, glucose transporter 1; HBV, hepatitis B virus; HCV, hepatitis C virus; miRNA, microRNA; OGT, O‐GlcNAc transferase; SAMHD1, sterile alpha motif and histidine/aspartic acid domain‐containing protein 1; ST8SIA3, ST8 alpha‐N‐acetyl‐neuraminide alpha‐2,8‐sialyltransferase 3.

As for the biological functions of O‐glycosylation in HBV, researchers have shown that GALNT7 plays an essential role in HBV life cycle through initiating O‐linked glycosylation through transferring O‐GalNAc to viral proteins in the Golgi apparatus (Figure 1)^14^. Knocking down GALNT7 could significantly decrease the levels of HBV DNA and HBsAg^14^. In addition, ST8 alpha‐N‐acetyl‐neuraminide alpha‐2,8‐sialyltransferase 3 (ST8SIA3), a sialyltransferase, is also required for the secretion of HBV DNA and HBsAg, suggesting that targeting GALNT7‐mediated O‐GalNAc could hold promise as a therapeutic target for HBV (Figure 1)^14^.

O‐GlcNAcylation is also crucial for HBV. It has been observed that HBV infection enhances the level of glucose transporter type 1 (GLUT1), leading to elevated glucose uptake and protein O‐GlcNAcylation. This, in turn, suppresses HBV replication. Conversely, diminished O‐GlcNAcylation levels have been found to promote autophagosome formation through several mechanisms. These include inducing ER stress, inhibiting Akt/mTOR signaling, and impeding autophagosome–lysosome fusion^15^, ^16^. Besides, it was found that the inhibition of the HBP pathway increased HBV replication^17^. Mechanistically, it was pinpointed that sterile alpha motif and histidine/aspartic acid domain‐containing protein 1 (SAMHD1) is O‐GlcNAcylated at Ser93^17^. As a deoxynucleotide triphosphate triphosphohydrolase (dNTPase), SAMHD1 functions by converting deoxynucleoside triphosphates (dNTPs) into their corresponding deoxynucleosides and inorganic triphosphates^18^. However, the incorrect processing of host nucleic acids results in the sustained activation of the innate immune system, which ultimately contributes to the persistent activation of the immune system. O‐GlcNAcylation has been found to stabilize SAMHD1, promote its tetramer formation, and upregulate its dNTPase activity^17^, again restricting viral DNA synthesis. Taken together, at the early stage of HBV infection (before progression into HCC), O‐GlcNAcylation might attenuate HBV replication by downregulating the autophagy pathway^16^ or upregulating the interferon (IFN)‐induced SAMHD1 in the host cells (Figure 1)^17^.

Moreover, initially discovered in yeast, autophagy is a conserved process to engulf damaged proteins or cellular organelles during famine or stress^19^. Upon HBV infection, the autophagic process is activated, which may hijack the autophagic membrane system to promote viral replication and release^20^, ^21^. During the late stages of liver diseases (e.g., HCC), however, autophagy might lead to cell death and thus curb disease progression. Therefore, autophagy in HBV is a double‐edged sword whose role is perplexing and only nascently understood. Adding to the complex picture is protein O‐GlcNAcylation, which also rises or falls in response to nutrient status, especially glucose concentration. Research findings have revealed that decreased glucose levels may potentially enhance HBV replication, possibly via the autophagy pathway mediated by mTOR^22^. Further attenuation of the cellular O‐GlcNAc levels by utilizing an OGT inhibitor (OSMI‐1) or small interfering RNA (siRNA) targeting OGT could promote HBsAg protein levels and HBV replication possibly by upregulating the autophagy pathway (Figure 1)^16^. This is consistent with the notion that low glucose might decrease UDP‐GlcNAc biosynthesis and thus high levels of autophagy, and OGT negatively regulates HBV infection by suppressing autophagy. However, it is known that the cellular O‐GlcNAc levels can rise during famine or fasting^23^. Therefore, studies like this need to closely monitor the overall O‐GlcNAcylation levels.

In a nutshell, both O‐GalNAc modifications on viral proteins and O‐GlcNAc modifications on host proteins play crucial roles in the life cycle of HBV. GALNT7‐mediated O‐GalNAc prompts HBsAg and HBV DNA secretion, while the cellular O‐GlcNAc suppresses the HBV replication and infection. However, whether there are other biological functions of O‐glycosylation in the HBV life cycle is still not certain, which is worthy of being further studied.

### Herpesvirus

Herpesviruses, one group of double‐stranded DNA viruses, give rise to diverse clinical manifestations and diseases. There are eight herpesviruses that impact human health, and they can be categorized into three subfamilies: *Alphaherpesvirinae*, *Betaherpesvirinae*, and *Gammaherpesvirinae*. *Alphaherpesvirinae* consists of human herpes simplex virus types 1 and 2 (HSV‐1 and HSV‐2), bovine herpesvirus (BOHV), pseudorabies virus (PRV), varicella‐zoster virus (VZV), equine herpesvirus (EHV), duck plague virus (DPV), herpes simian B virus, and Marek's disease virus (MDV). *Betaherpesvirinae* consists of species of cytomegaloviruse (CMV). *Gammaherpesvirinae* consists of several genera commonly known as Epstein‐Barr virus (EBV), Kaposi's sarcoma‐associated herpesvirus (KSHV), lymphocryptovirus found in rhesus monkeys, *Herpesvirus papio* found in baboons, *Herpesvirus saimiri*, and *Herpesvirus ateles*. In this review, we will elaborate on HSV‐1 and HSV‐2 and touch upon some other herpesviruses.

**Figure 2:**
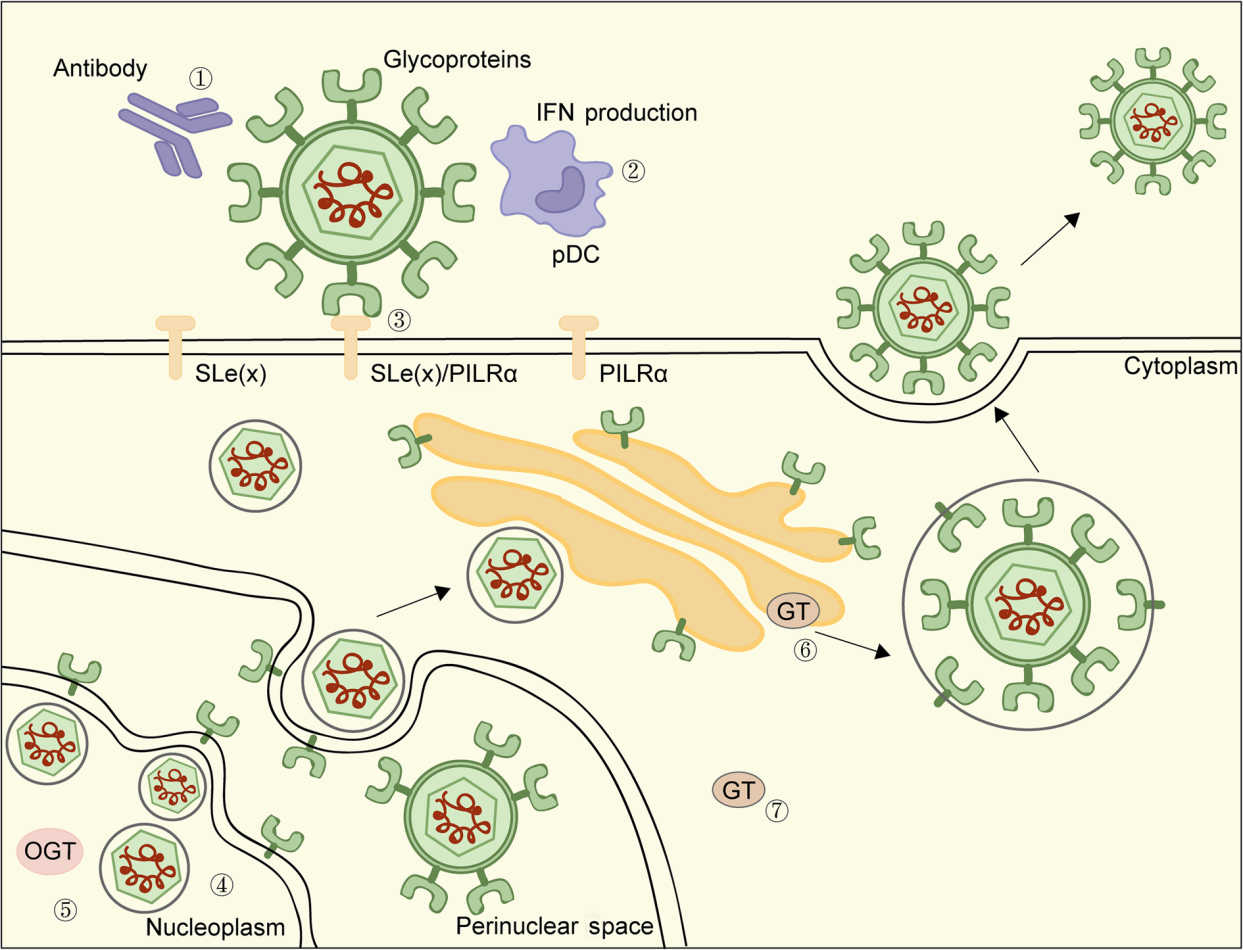
Diverse functions of O‐glycosylation of viral proteins from herpesvirus. (1) Epstein–Barr virus gb350/BOHV‐4 gb180: antibody evasion. (2) Pseudorabies virus gD: activating pDC and inducing interferon. (3) HSV‐1 gC: viral attachment and diffusion; HSV‐1 gB: PILRα‐dependent viral entry. (4) KSHV ORFs: DNA synthesis and replication. (5) HSV‐1, HSV‐2, cytomegaloviruses: OGT takes part in the late stage of viral replication. (6) BOVH‐4, a homolog of C2GnT‐M: modulating GT's activity. (7) HSV‐1 gC: activating the host's GT genes. BOVH4, Bovine Herpesvirus 4; C2GnT‐M, cellular core 2 protein β‐1,6‐N‐acetylglucosaminyltransferase‐mucintype; gD, glycoprotein D; GT, glycosyltransferases; KSHV, Kaposi's sarcoma‐associated herpesvirus; pDC, plasmacytoid dendritic cell; PILRα, paired immunoglobulin‐like type 2 receptor α; SLe(x), sialyl‐Lewis X.

HSV‐1 and HSV‐2, members of the *Alphaherpesvirinae* subfamily, are highly prevalent worldwide^17^. The crucial step for HSV‐1 to enter host cells is the interaction between cell surface receptors and the virion envelope GPs. Glycoprotein C (gC), which contains a high density of O‐glycosylation, facilitates the attachment of HSV‐1 to susceptible host cells by binding to glycosaminoglycans (GAGs) on the cell surface. The virus‐GAG interaction can be regulated by modifying the affinity, type, and number of GPs involved^17^, ^24^. However, the dynamics of virus–GAG interactions remain poorly understood. Recent research has highlighted the significant role of viral mucin‐like domains (MLDs) in HSV‐1 gC in controlling the initial interactions between the virus and the glycocalyx of the target cells^25^. These interactions facilitate the virus's navigation through the glycocalyx and enhance its ability to penetrate the target cell at an early stage. When certain herpesviruses activate the transcription of host fucosyltransferase (FUT) genes, which encode a fucosyltransferase involved in the synthesis of sialyl‐Lewis X (sLe[x]), the selectin receptor sLe(x) can be induced. The mucin domains of HSV‐1 gC‐1 play a role in facilitating the expression of selectin ligands, including sLe(x) and other larger O‐linked glycans, in cell types that lack endogenous mucin domain‐containing GPs. This optimization of O‐glycan expression helps activate the appropriate host glycosyltransferase genes (Figure 2)^26^. Additionally, the paired immunoglobulin‐like type 2 receptor α (PILRα), an entry receptor for HSV‐1, has the ability to interact with sialylated O‐glycans present on the envelope glycoprotein B (gB) of HSV‐1^27^, ^28^. Researchers have been demonstrated that gB O‐glycosylation, probably at Thr53 and Thr480 of gB, could prompt PILRα‐dependent viral entry and enhance viral replication and virulence^29^. Interestingly, recent studies have shown that the N‐terminus of glycoprotein K (gK) plays a functional role in association with the gB–PILRα protein complex. This association regulates the fusion of the viral envelope with cellular membranes during viral entry and facilitates virus‐induced cell‐to‐cell fusions^30^. Furthermore, both glycoprotein D 1 (gD1) and gD2 have been found to undergo O‐glycosylation, with gD2 exhibiting higher levels of O‐glycosylation than gD1^31^. Additionally, glycoprotein I (gI) of HSV‐1 contains a unique polymorphic tandem‐repeated mucin region that can undergo modification by O‐GalNAc monosaccharides, specifically the Tn antigen^30^. Furthermore, the biosynthesis of O‐glycans on HSV gC is strictly dependent on host galactosyltransferases^31^. Besides, research has demonstrated that GALNT1 has the ability to catalyze the addition of O‐GalNAc to immature forms of herpesvirus GPs, including pgC, pgD, and gA‐pgB^32^.

Apart from its role in modifying viral proteins with O‐GalNAc, OGT also participates in HSV replication. This process entails the sequential expression of immediate‐early, early, and late genes. Researchers utilized human foreskin fibroblasts (HFFs) and used the OGT inhibitor OSMI‐1 to inhibit the catalytic function of OGT. Their findings revealed that treatment with OSMI‐1 led to a decrease in the production of HSV‐1, HSV‐2, and cytomegalovirus, potentially impacting the latter stages of viral replication. This indicates that OGT could have a critical function in the effective replication of these viruses^33^. The use of siRNA to target OGT was found to reduce the immediate‐early gene expression of HSV, suggesting that OGT also functions as a catalytic enzyme in the life cycle of HSV^33^. One of the targets of OGT is host cell factor 1 (HCF‐1), which forms a complex with VP16 and octamer‐binding protein 1 (Oct‐1) to regulate immediate‐early gene expression by binding directly to their promoters and recruiting other transcriptional factors. In addition to O‐GlcNAcylating HCF‐1, OGT has been discovered to moonlight as a protease that cleaves HCF‐1^34^, ^35^. Cleaved HCF‐1 differently binds with transcription activators or corepressor four‐and‐a‐half LIM domain‐2 (FHL2)^36^. Therefore, OGT might promote the replication of HSV via distinct routes.

O‐glycans also impact PRV, which belongs to the *Alphaherpesvirinae* subfamily. Plasmacytoid dendritic cells (pDCs) are important innate immune cells that respond to viral infections by producing substantial amounts of antiviral type I IFN. In the case of PRV infection, O‐glycosylation, in conjunction with the gD envelope GP, plays a critical role in activating pDCs. Specifically, the elimination of both O‐ and N‐glycans from PRV virions significantly reduces the production of IFN. However, interestingly, the production of IFN increases when only N‐glycans are removed. The interplay between O‐glycans, gD envelope GP, and pDC activation highlights their significance in the immune response against PRV infection^37^.

KSHV relies on the replication and transcription activator (RTA), also known as K‐RTA, which is an immediate‐early protein responsible for both KSHV replication and reactivating latent KSHV, a member of the *Gammaherpesvirinae* subfamily^38^. K‐RTA is phosphorylated at Ser634 and Ser636 by host CDK9 proteins and required for the full reactivation of KSHV lytic cycle^39^. K‐RTA is also O‐GlcNAcylated at Thr366 and Thr367 to repress its transactivation activity^39^. Mechanistically, O‐GlcNAcylated K‐RTA would increase binding with poly (ADP‐ribose) polymerase 1 (PARP1), a transferase that modifies proteins with polymer ADP‐ribose (PAR)^39^, while PARP1 acts as a suppressor of K‐RTA (Figure 2)^40^. It remains unclear whether K‐RTA is subject to modification by PARP1, or whether there is a crosstalk between K‐RTA O‐GlcNAcylation and PARylation. Treatment of KSHV‐infected cells with short hairpin RNA targeting OGT (shOGT) has been shown to result in viral reactivation^39^, suggesting that O‐GlcNAcylation may serve as a host antiviral mechanism. This also suggests that O‐GlcNAcylation is involved in regulating KSHV latency and hints at the possibility that targeting OGT could serve as a strategy to disturb viral latency and boost host immune responses against the virus. In a comprehensive screening, all 85 KSHV ORFs were analyzed for O‐GlcNAcylation, and it was discovered that 18 proteins were glycosylated^41^. These glycosylated proteins were found to be enriched in the processes of DNA synthesis and replication. This also suggests that O‐GlcNAcylation may play a regulatory role in these critical cellular processes during KSHV infection, potentially contributing to viral replication and persistence. However, due to limitations in MS technology at that time, the specific O‐GlcNAcylated sites on these proteins could not be determined. Nevertheless, it is plausible that OGT plays a pivotal role in KSHV propagation, and further investigation into the functions of these O‐GlcNAcylated proteins would provide valuable insights into the molecular mechanisms underlying KSHV infection and replication.

In terms of O‐glycans on Herpesvirus envelope proteins, such as VZV, CMV, and EBV, with a “bottom‐up” MS‐based technique for mapping O‐glycosylation sites, researchers have demonstrated that there were widespread O‐glycans in the regions of viral envelope proteins, which possessed conserved patterns on distinct homologous proteins^42^. Furthermore, they found that infected cells maintained their capacity to elongate most of the surface glycans and such elongated O‐glycans could prompt HSV‐1 to produce markedly higher viral titer. The envelope proteins of VZV have been shown to be highly immunogenic, and one of the most abundantly expressed proteins is glycoprotein E (gE). Besides, in terms of other herpesviruses, the highly O‐glycosylated gp350, the homolog of the BoHV‐4 gp180, takes part in the binding of EBV to B cells and serves as a target for neutralization. In comparison to other virions, it was observed that the removal of O‐glycans from these virions made them more susceptible to neutralization by anti‐BoHV‐4 serum. This observation indicates that O‐glycans on the gp180 GP act as a crucial glycan shield for protecting vulnerable viral epitopes^43^. Additionally, it has been reported that BoHV‐4 has a *Bo17* gene that encodes a homolog of the cellular core 2 protein β‐1,6‐N‐acetylglucosaminyltransferase‐mucin type (C2GnT‐M), which is critical for synthesizing complex O‐glycans. As a result, researchers suggest that the splicing of the *Bo17* gene allows BoHV‐4 to regulate the overall level of core 2 branching activity within infected cells. This, in turn, may impact the activity of glycosyltransferases found in the Golgi apparatus^44^.

In summary, O‐GalNAc and O‐GlcNAc modifications play pivotal roles in the life cycle of herpesviruses. Specifically, in HSV, O‐GalNAc on viral proteins is involved in viral entry, diffusion, and replication. Additionally, O‐glycans play a crucial role in activating innate immune cells, evading antibody responses, and promoting the production of higher viral titers. Furthermore, in HSV, cellular O‐GlcNAc, mediated by OGT, may promote viral replication through distinct mechanisms, including O‐GlcNAcylation of HCF‐1 and its subsequent cleavage. In the context of KSHV, O‐GlcNAc actively suppresses the reactivation of the KSHV lytic cycle and plays a pivotal role in the host's defense mechanism against the invading virus. Moreover, the cellular O‐GlcNAc in KSHV is also implicated in DNA synthesis and replication. These results highlight the significance of O‐GalNAc and O‐GlcNAc modifications in the regulation of herpesvirus infections and provide insights into their impact on viral replication and host–virus interactions.

### Human papillomavirus (HPV)

HPV has been demonstrated to cause cervical cancer and head and neck squamous cell carcinoma (HNSCC). Intriguingly, HPV16 oncogene E6 or E6/E7 would upregulate both mRNA and protein levels of OGT and increase cellular O‐GlcNAcylation^45^. C‐Myc is an oncoprotein against HPV, whose stability and O‐GlcNAcylation on Thr58 can be enhanced by E6^45^. Knockdown of OGT or using OGT inhibitors would suppress tumorigenesis, probably through decreasing c‐Myc protein abundance^45^. HPV infection also causes the increased stability of Unc‐51‐like autophagy activating kinase 1 (ULK1) in HNSCC cells, probably via enhanced O‐GlcNAcylation on Ser409 and Ser410, and inhibition of the chaperon‐mediated autophagy, which consequently enhances HPV‐induced autophagy to promote the viral clearance^46^. Interestingly, high levels of ULK1 correlate with HPV‐positive HNSCC patient survival, suggesting that ULK1 O‐GlcNAcylation could be the underlying mechanism. HPV also causes the metastatic spread of lung cancer, while OGT increases the levels of proteins E6 and E7 in both HeLa cells and lung xenografts^47^. Short hairpin RNA (shRNA) targeting OGT would reduce tumor formation by reducing protein levels of E6 and E7^47^. These data suggest potential positive feedback between HPV E6 and OGT.

Interestingly, there is evidence linking HPV infection with the downregulation of OGT. It has been reported that the viral protein E6 stimulates the activity of E3 ubiquitin ligase E6AP, which promotes viral survival^48^, and E6AP has also been implicated in the proteasome‐mediated degradation of OGT, resulting in a decrease in total levels of O‐GlcNAcylation^49^. We speculate that both scenarios may be true, depending on the amount of E6 protein transduced into the cells. The disease‐causing mechanism of HPV is still not fully understood, but it is possible that HPV induces higher O‐GlcNAcylation levels at the early step of infection, while OGT degradation may occur at later stages. Furthermore, it is worth noting that HPV may impact multiple cell types, such as HEK293 cells, HeLa cells, and HNSCC cells, through distinct mechanisms. Further research is needed to elucidate the precise mechanisms by which HPV modulates O‐GlcNAc levels and its implications in HPV‐associated diseases.

In summary, O‐GlcNAc modifications play a crucial role in the life cycle of HPV. OGT has been implicated in enhancing tumor formation and tumorigenesis associated with HPV infection. Moreover, the levels of O‐GlcNAcylation may be influenced by the amount of transduced E6 protein, which could impact viral clearance or viral survival. These findings highlight the significance of O‐GlcNAc modifications in HPV pathogenesis and suggest their potential as targets for therapeutic interventions.

## THE ROLES OF O‐GLYCOSYLATION IN RNA VIRUSES

### Filovirus

Ebola virus (EBOV) and Marburg virus (MARV), members of the *Filovirus* family, are highly pathogenic viruses in humans, which can cause serious hemorrhagic diseases with a high mortality rate^50^. The interaction among nucleoprotein (NP) and virion‐associated proteins (VP35 and VP24) is essential for the formation of EBOV particles, specifically the outer layer of the nucleocapsids^51^. Recent studies have revealed the involvement of O‐glycosylation and sialylation in the formation of the NP‐VP35 complex during capsid formation in EBOV and its related viruses. O‐Glycosylated NP is able to coprecipitate with VP35, whereas deglycosylated NP does not interact with VP35^52^. This finding holds great potential for the development of antiviral therapies and vaccines targeting these viruses. Understanding the role of O‐glycosylation and sialylation in the interaction between NP and VP35 during capsid formation could provide valuable insights for the design and development of effective interventions against EBOV and its related viral infections. These findings open up new avenues for the development of antiviral strategies and hold promise for future advancements in combating these deadly viruses^52^. Treatment with deglycosylation enzymes or α2‐3,6,8,9 neuraminidase alone resulted in altered migration of NP on the gel, suggesting the presence of sugars that contain terminal sialic acid. These findings indicate the involvement of sialic acid‐containing sugars in the glycosylation of NP. The identification and characterization of these specific sugar modifications provide valuable insights into the glycosylation patterns of NP and their potential functional significance. Further investigation into the role of sialic acid‐containing sugars in NP glycosylation could advance our understanding of viral pathogenesis and aid in the development of antiviral strategies targeting EBOV and its related viruses.

Besides, it is known that the full‐length GP of EBOV can be cleaved by furin into two subunits: GP1 and GP2. The MLD, one of the domains in GP1, plays a crucial role in virus attachment and entry. Moreover, glycosylation is vital to form glycan shield from neutralization by serum antibodies. In this context, researchers have directed their attention toward O‐glycosylation in the MLD, where they have discovered a multitude of O‐glycans. These include a combination of truncated Tn‐antigen and extended, sialylated core 1 and 2 structures. Additionally, they have identified several O‐glycosylation sites outside the MLD of EBOV GP, such as Thr280 and Thr206^53^. Interestingly, upon infection of host cells with EBOV, there was a significant decrease in endothelial cell adhesion induced by the surface GP. Emily et al. found that GALNT1 was essential for extended O‐glycans to exert the effect of deadhesion^54^. Direct comparisons between ppGalNAc‐T WT cells transfected with GP and GALNT1 depleted cells revealed a notable decrease in nonadherent cells in the latter. Specifically, it was observed that the adhesion activity of GP relied on O‐glycosylation sites within the MLD (segment 329–345)^54^.

In addition to EBOV, studies on the GP of MARV have identified the presence of O‐glycans with galactose‐β(1–3)‐N‐acetylgalactosamine disaccharide units. These findings were obtained through a series of experiments that assessed the sensitivity of GP to endo‐α‐/vacetylgalactosaminidase and peanut agglutinin (PNA). The identification of these specific O‐glycan structures provides valuable insights into the glycosylation patterns of the MARV GP. Further investigation into the functional significance of these O‐glycans during MARV infection could enhance our understanding of the viral pathogenesis and potentially inform the development of targeted antiviral strategies^55^.

In summary, O‐GalNAc modifications on EBOV proteins play multiple roles in the viral life cycle. They contribute to the amplification of viral particle formation, virus attachment, and virus entry. Additionally, O‐glycans on EBOV are involved in forming the glycan shield, helping the virus evade antibody neutralization. However, these O‐glycans may also lead to the loss of endothelial cell adhesion. In the context of filoviruses, more research is needed to further understand the functions of O‐GlcNAc modifications in the viral life cycle. Investigating the impact of O‐GlcNAc modifications could provide valuable insights into the mechanisms underlying filovirus infections and potentially lead to novel therapeutic strategies.

### Hepatitis C virus (HCV)

Similar to HBV, HCV is also associated with HCC. Claudin‐1 plays a critical role in the entry of HCV into the hepatocytes after viral binds to the receptor CD81, a member of the tetraspanin integral membrane protein family^56^. When it comes to the assembly and disassembly of claudins within tight junctions, phosphorylation at the C‐terminal tail of claudins plays a crucial role. Interestingly, researchers have discovered Yin Yang sites (Ser192, Ser205, Ser206) on which there could be an interplay between phosphorylation and O‐GlcNAc modification. They have also pinpointed O‐GlcNAc modification sites on claudin‐1 and have demonstrated that site mutations can prevent its phosphorylation. This, in turn, disrupts the binding of claudin‐1 with other cytoplasmic proteins and reduces its effectiveness in facilitating viral entry during HCV infection (Figure 1)^57^.

Moreover, elevated levels of OGT expression have consistently been observed in liver tissues of patients in clinical samples. Interestingly, there is no significant correlation between OGT expression and HCV RNA levels. Notably, the upregulation of OGT in HCV‐induced liver disease and cancer seems to be predominantly influenced by inflammation and fibrosis rather than being directly induced by HCV infection itself. This indicates that OGT might contribute to the advancement of HCV‐related liver disease through these particular mechanisms. In a recent systematic screening study aimed at identifying miRNAs that modulate HCV, miR‐501‐3p and miR‐619‐3p were discovered as novel regulators. These miRNAs may have potential implications in the regulation of HCV infection and could serve as targets for future therapeutic interventions^58^. Both miRNAs could affect the later life cycle of HCV assembly and release, and miR‐501‐3p more robustly regulated the posttranscriptional control of OGT^58^, which may further influence HCV assembly, release, and infectivity.

In conclusion, O‐GlcNAc modifications play an important role in HCV entry, and a higher expression of OGT, has been observed in HCV patients. However, the precise role of high OGT expression in liver diseases associated with HCV infection remains unclear. It is evident that OGT can modulate the assembly, release, and infectivity of HCV. More research is needed to further understand the functional implications of O‐GlcNAc modifications and the specific impact of elevated OGT expression in HCV‐related liver diseases. These investigations will provide valuable insights into the underlying mechanisms and potential therapeutic targets for HCV infection and its associated liver pathologies.

### Human immunodeficiency virus‐1 (HIV‐1)

HIV‐1, a retrovirus, poses a significant threat to the human immune system by targeting helper T lymphocytes, which can result in increased susceptibility to various bacterial infection and tumorigenesis. The envelope glycoprotein (Env) trimer of HIV‐1 plays a critical role in entry and infection. This trimeric structure consists of two essential subunits: the surface subunit, gp120, and the transmembrane subunit, gp41. Together, these two subunits facilitate the attachment of HIV‐1 to host cells and mediate the fusion, enabling the virus to enter and infect target cells. Gaining a comprehensive understanding of the structure and functionality of the HIV‐1 Env GP is crucial for devising effective strategies to combat HIV‐1 infection and mitigate its detrimental effects on the immune system.

The identification of O‐glycosylation sites on the HIV‐1 Env gp has been a complex and evolving process, highlighting the challenges in viral O‐glycan analysis. Initially, conventional lectin‐binding assays suggested that HIV‐2 and Simian Immunodeficiency Virus (SIVmac239) were O‐glycosylated at the V1 domain of gp120, while HIV‐1 was not^59^. However, subsequent studies using secreted gp120 protein revealed O‐glycan modifications at the C‐terminal Thr499^60^, ^61^. Interestingly, when gp120 was analyzed from purified virions, no O‐glycan was found on Thr499 on the virion surface^62^. A recent study utilizing HIV‐1 virions derived from patients and secreted gp120 revealed that Env gp120 is indeed subject to O‐glycosylation on the Variable 1 (V1) domain. This particular glycosylation has the potential to provide a protective shield for the virus against neutralizing antibodies that target the V3‐glycan region, thus aiding in viral evasion and escape^63^. In another investigation using recombinant gp120 proteins, a combination of EThcD‐sceHCD‐MS/MS was used to analyze the N‐ and O‐glycosylation of gp120. By using this approach, the identification of glycopeptides was significantly enhanced, resulting in a more comprehensive array of fragment ions for conducting site‐specific glycosylation analysis. Notably, this method proves particularly advantageous for precise and reliable assignment of O‐glycosites^64^. Consequently, five O‐glycosites with attached glycans (Thr60, Ser132, Ser308, Thr382, and Thr419) were unambiguously assigned in heavily glycosylated gp120^64^. However, it remains uncertain whether these O‐glycosylated forms of gp120 exist on native virions. We anticipate that the exploration of HIV‐1 glycosylation will continue to evolve as ongoing technical advancements are made. However, it is crucial to exercise extreme caution when working with recombinant proteins in these studies. The intricacies of HIV‐1 glycosylation necessitate careful interpretation of the results, considering the potential differences between recombinant proteins and native viral GPs. To ensure accurate and reliable findings, it is important to use rigorous methodologies and validate the results using multiple approaches, including analysis of native virions. By approaching HIV‐1 glycosylation research with meticulousness and critical evaluation, we can advance our understanding of this important aspect of the virus and its implications for pathogenesis and immune responses.

The role of glycosylation in viral infectivity has been a subject of investigation. Specifically, the impact of O‐glycosylation on the highly conserved C‐terminus of gp120 has been studied. Researchers have demonstrated that O‐glycosylation is not essential for the natural replication cycle of HIV‐1, as the virus retains full infectivity even in a knockout cell line lacking O‐linked carbohydrates. However, it is plausible that in vivo, natural target cells for HIV may still complete O‐glycan attachment to potentially enhance infectivity further. Although not necessary for the virus's basic replication, O‐glycosylation might contribute to specific aspects of viral entry or interactions with host factors, highlighting the complex interplay between glycosylation and viral infectivity. More research is needed to further elucidate the functional significance of O‐glycosylation in the context of HIV‐1 infection and its potential implications for viral pathogenesis^65^.

Regarding therapeutic applications, previous studies have shown that the glycan shield of HIV‐1 can function as a protective barrier, effectively safeguarding the Env from the humoral immune response. This shielding mechanism reduces the immunogenicity of the virus and limits the accessible protein surface for antibody binding^66^. Building upon this characteristic, researchers have explored the generation of synthetic glycopeptides by introducing O‐GlcNAc glycosylation to HIV peptides through glycoengineering. The glycosylated peptides exhibited reduced susceptibility to T cell recognition by interfering with human leukocyte antigen binding or T cell receptor recognition^67^. Both in silico simulations and ex vivo studies have provided evidence that glycosylation has the potential to mask viral recognition by T cells, suggesting that glycoepitopes could modify the host T cell immune response^67^. These findings offer a proof‐of‐concept analysis highlighting the potential of glycoepitopes to modulate T cell immunity and may pave the way for the future development of novel therapeutic strategies targeting HIV‐1.

Interestingly, O‐GlcNAcylation of host proteins during HIV infection has also been identified. Approximately a decade ago, a proteomic screening method was utilized, which involved a combination of fluorescent two‐dimensional (2‐D) gel‐based technique and enzymatic pretreatment of the virus using Peptide:N‐Glycosidase F (PNGase F). Subsequently, fluorescent 2‐D gel or 2‐D gel western blot analysis was used for analysis^68^. The results of this study not only revealed the presence of typical N‐glycosylated proteins but also identified an O‐GlcNAcylated host protein, specifically HLA‐DR^68^. However, due to the limitations of MS technology at that time, it was not feasible to further investigate the specific modification site or function of this O‐GlcNAcylated host protein. Nonetheless, this pilot study provided an intriguing indication that host proteins may undergo O‐GlcNAc modification in response to viral infection. This finding opens up avenues for further elucidating the function and significance of O‐GlcNAcylation in the context of host–virus interactions during HIV infection.

In summary, concerning HIV‐1, O‐GalNAc modifications on viral proteins could play a role in shielding the virus against neutralizing antibodies, contributing to immune evasion, and potentially enhancing infectivity. However, the precise functions and implications of cellular O‐GlcNAc modifications in the context of HIV‐1 infection have not been fully elucidated. Further research is needed to comprehensively understand the functional significance of O‐GlcNAc modifications on both viral and cellular proteins during HIV‐1 infection. By unraveling these mechanisms, we can gain valuable insights into the interplay between O‐GlcNAc modifications and HIV‐1 pathogenesis, potentially opening avenues for novel therapeutic strategies targeting these processes.

### Human T‐cell lymphotropic virus (HTLV‐1)

HTLV‐1, a human retrovirus, is associated with adult T‐cell leukemia, which is characterized by malignant CD4^+^ T lymphocyte over‐production^69^. A critical oncoprotein in HTLV‐1‐mediated tumorigenesis is the viral Tax protein that regulates both viral replication and viral pathology. Tax not only promotes its own transcription and expression but also enhances the binding of CAMP‐response element binding protein (CREB) to the U3 region of the virus^69^. Tax regulates OGA from many perspectives. First, in T cells transformed by HTLV‐1, there is an observed increase in both the mRNA and protein levels of OGA. Second, the overproduced OGA turns out to be a less active form of OGA. Third, when transfected into Tax‐negative cells, Tax would increase cellular O‐GlcNAcylation by downregulating OGA activity. Fourth, both OGT and OGA are recruited to the viral 5′‐untranslated region. Mechanistically, it is plausible that Tax might impede OGA activity, resulting in elevated CREB O‐GlcNAcylation and promoting the recruitment of CREB to the long terminal repeat (LTR). As a result, Tax's role in LTR transactivation is enhanced, establishing an advantageous environment that facilitates the establishment of a positive feedback loop. It would be interesting to test whether Tax itself is O‐GlcNAcylated.

In conclusion, O‐GlcNAc modifications play a significant role in HTLV‐1 infection, potentially influencing viral replication and host cell binding. However, there are still many other aspects and roles of O‐GalNAc and O‐GlcNAc modifications in relation to this virus that require further investigation. Future studies are needed to explore and understand the full extent of these modifications and their implications in HTLV‐1 infection. By unraveling the complexities of O‐GalNAc and O‐GlcNAc modifications, we can gain a deeper understanding of the molecular mechanisms underlying HTLV‐1 pathogenesis and potentially identify novel therapeutic targets for combating this infection.

### Influenza virus (IV)

The *Orthomyxoviridae* family includes IVs, which are divided into four genera: influenza A virus (IAV), influenza B virus (IBV), influenza C virus (ICV), and influenza D virus (IDV). IAV is the most clinically significant type among them and is classified into different categories based on the type of surface GPs, namely hemagglutinin (HA) and NA. Currently, there are 18 HA subtypes and 11 NA subtypes in humans, while birds have 16 HA and 9 NA subtypes. Human and bird IVs also differ in their N‐glycosylation types—human HA utilizes α,6‐linked sialic acid, whereas bird HA uses α2,3‐linked sialic acid^3^. Studies of IV exemplify what glycosylation studies could bring to modern medicine, as three antiflu drugs (oseltamivir, zanamivir, and peramivir) were developed to target and inhibit the enzymatic function of NA, thus blocking viral escape from the infected cells^70^.

The presence of O‐glycosylation on secreted IA proteins is an emerging discovery. A study in 2015 found that the secreted HA1 protein of IAV H1N1 Puerto Rico contains O‐glycosylation at a conserved Thr residue which is located 12 amino acids from the C‐terminus. Additionally, it was discovered that the HA1 protein of IAV H5N1 Thai is O‐glycosylated at a Thr residue situated 16 amino acids from the C‐terminus. However, the biological significance of these O‐glycosylations remains unclear^62^. Notably, both O‐glycosylations are characterized by the presence of disialylated core 1 glycans, displaying related core 1 and core 2 structures^62^. Given that this O‐glycosylation site is conserved in HIV‐1 (gp120) and simian immunodeficiency virus from rhesus macaques (SIVmac) (gp120), it would be intriguing to investigate its potential functional role in these viral proteins. Further research in this field could provide valuable insights into the significance of O‐glycosylation in the context of IVs and other related viral infections.

In addition, IV triggers O‐glycosylation in the host cells. Following an IAV infection, mucin‐type proteins are quickly generated on the epithelial surfaces of the respiratory tract. Nakamura and colleagues were the first to show that GALNT3 mRNA is elevated during the early stage of IAV infection. This elevation results in heightened mucin production and improved IAV replication within the infected cells^71^. Conversely, GALNT3 plays a critical role in inhibiting inflammation during IAV infection by impeding the translocation of phosphorylated P65 into the nucleus^72^.

It has been established that IV HA1 interacts with the natural killer (NK) cell receptor, NKp46, and facilitates the lysis of IV‐infected cells. O‐glycosylation has been detected on Thr125 of NKp46, and the O‐glycan sequences have been identified to mediate the interaction between rNKp46 and IV H1N1. This interaction may involve the branched alpha2,3‐sialylated O‐glycoform Neu5NAcalpha2,3‐Galbeta1,4‐GlcNAcbeta1,6[Neu5NAcalpha2,3‐Galbeta1,3]GalNAc^73^. α‐Dystroglycan (α‐DG) serves as a receptor in the basement membrane and undergoes extensive glycosylation. It is cleaved by furin, releasing an N‐terminal domain referred to as α‐DGN. The MLD of α‐DGN undergoes O‐glycosylation, a process mediated by large xylosyl‐ and glucuronyltransferase (LARGE1). Recent research indicates that α‐DGN plays a protective role in inhibiting IAV proliferation. Mice lacking α‐DGN showed higher viral titers, underscoring the significance of α‐DGN in regulating IAV infection^74^.

O‐GlcNAc is also involved in IAV‐inflicted host cells. During IAV infection, viral components might cause downstream humoral responses. The most known response is “cytokine storm”^75^. During immune activation, glucose uptake increases in the inflammation response^76^, but it is unclear whether OGT is involved in the process or OGT has specific targets in the due course. Recently, it was demonstrated that IFN regulatory factor‐5 (IRF5)‐mediated pathway was essential for IAV‐induced cytokine storm^77^. More specifically, IAV induced IRF5 O‐GlcNAcylation at Ser430, leading to subsequent K63‐ubiquitination of IRF5^77^, which was critical for IRF5 function^78^. Furthermore, IRF5 O‐GlcNAcylation mediated the nuclear translocation of IRF5 and cytokine production^77^. Mouse studies and clinical sample analyses from IAV patients correlated with the biochemical results and both showed blood glucose upregulation with high levels of cytokines, suggesting that enhanced HBP flux could be a countermeasure against IAV.

In summary, O‐GlcNAc modifications play a crucial role in the life cycle of IV. GALNT3 is implicated in enhanced viral replication and inflammation inhibition. Additionally, O‐glycans mediate the interaction between IV and rNKp46, an NK cell receptor. Furthermore, the O‐glycosylation of α‐DGN has been found to suppress IAV proliferation. Furthermore, studies have shown that the O‐GlcNAcylation of IRF5 can amplify the cytokine storm induced by IAV infection.

### Paramyxoviruses

The paramyxovirus is a single‐stranded RNA virus that primarily spreads via respiratory systems. O‐glycosylation has been observed in several paramyxoviruses, including Hendra henipavirus (HeV) and Newcastle disease virus (NDV). Additionally, O‐GlcNAcylation has been found to play a role in the infectivity of respiratory syncytial virus (RSV).

The HeVs are highly lethal zoonotic paramyxoviruses, causing severe disease outbreaks in both humans and livestock^77^. Like other paramyxoviruses, HeV relies on both a fusion (F) and attachment (G) protein to facilitate cell–cell fusions. Specifically concerning Henipavirus, multiple specific O‐glycan structures in a stalk domain of HeV G have been identified, playing a crucial role in the fusion‐triggering process. Jacquelyn and colleagues have unveiled several novel functions of paramyxovirus O‐glycans, including significant modulation of cell–cell fusion, effects on G/F interaction, G conformation, receptor‐induced G conformational changes, and most notably, F processing and F incorporation into pseudotyped virions^78^. Furthermore, a previously undocumented O‐linked glycopeptide (66AEEKITSALGSNQVVDR83) with two sialylated glycoforms (HexNex1Hex1NeuAc1 and HexNAc1Hex1NeuAc2) has been revealed in the stalk domain of the HA‐NA protein in NDV, with three potential sites of O‐linked glycosylation (Thr71, Ser72, and Ser76). This discovery may provide an opportunity to evaluate the significance of the O‐glycan in the stalk domain of HA‐NA protein^79^.

Moreover, in RSV, another paramyxovirus, OGT has been demonstrated to prompt viral RNA synthesis in the cytoplasmic inclusion bodies (IBs). Incidentally, in IBs, there are also two signaling proteins: p38 mitogen‐activated protein kinase and OGT^80^. By hijacking the OGT in IBs, RSV suppresses stress granule formation^80^. Oxidative stress such as arsenite treatment would disassemble large IBs into smaller bodies, suggesting that OGT is involved in the dynamic regulation of IB formation when facing environmental insults^80^. It would be interesting to identify OGT substrates in the IB and delineate its potential role in viral replication.

In summary, regarding HeV, O‐glycans play a significant role in modulating the fusion‐triggering process. However, the functional significance of O‐glycans on NDV is still not clearly assessed. It has been demonstrated that O‐GlcNAc modifications in paramyxoviruses can enhance viral RNA synthesis and modulate oxidative stress in RSV.

### Severe acute respiratory syndrome coronavirus 2 (SARS‐CoV‐2)

SARS‐CoV‐2, the virus responsible for the coronavirus disease 2019 (COVID‐19) pandemic, is capable of infecting multiple organs in the human body, including the respiratory, circulatory, nervous, and gastrointestinal systems. The association between the viral spike (S) protein and its receptor angiotensin‐converting enzyme 2 (ACE2) plays a critical role in facilitating the internalization of the virus into human cells.

**Figure 3:**
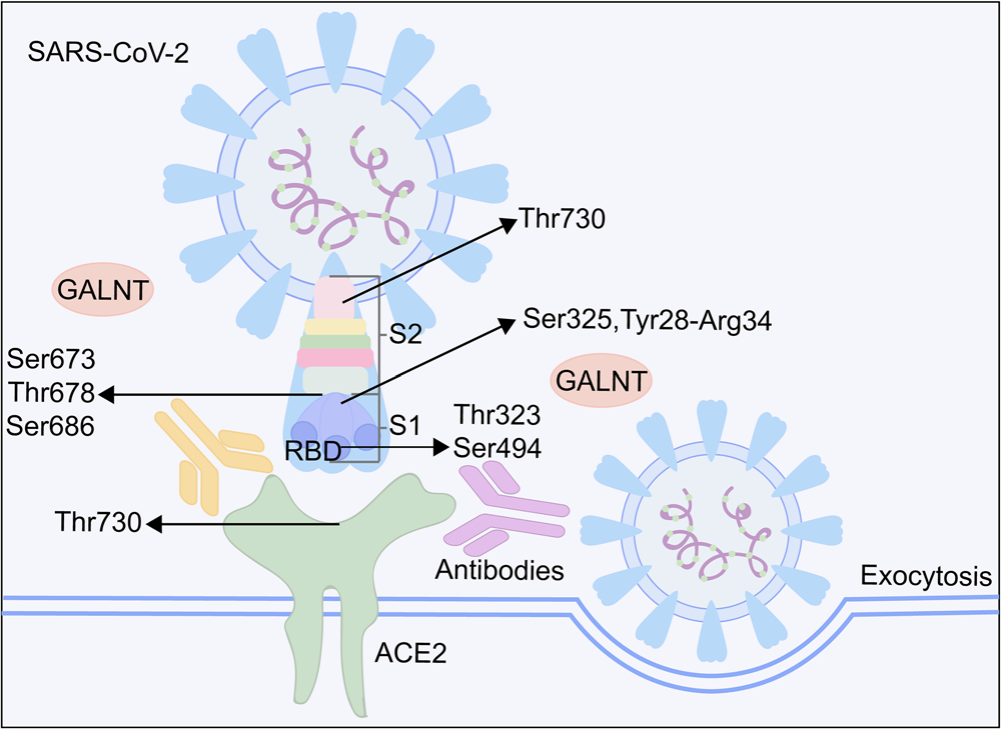
A diagram of O‐glycosylation sites on SARS‐CoV‐2 spike and ACE2, involving viral fusion and immune evasion. ACE2, angiotensin‐converting enzyme 2; RBD, receptor‐binding domain; SARS‐CoV‐2, severe acute respiratory syndrome coronavirus 2.

Investigators have discovered that both the SARS‐CoV‐2 S protein and its receptor‐binding domain (RBD) undergo extensive glycosylation, as depicted in Figure 3. Through the analysis of LC‐MS/MS data, the O‐glycosylation of the S1 and S2 subunits of the SARS‐CoV‐2 S protein was assessed. Through O‐glycoproteomic profiling, it has been discovered that O‐glycosylation occurs at positions Thr323 and Ser325 on the S1 subunit of the SARS‐CoV‐2 S protein^81^. Furthermore, the conventional RBD sequence contains an unpaired cysteine residue (Cys538) near the C‐terminus. This characteristic increases the likelihood of RBD constructs forming dimers when expressed in plant, insect, and mammalian cells. Interestingly, the presence of intact Thr323 O‐glycosylation is crucial for the expression and stability of the RBD as a monomer^82^. By mutating Thr323 into alanine, researchers were able to eliminate the primary O‐glycosylation site. As a result, they observed fewer dimers of the RBD. This mutation has the potential to significantly streamline the purification process during vaccine production and maximize the utilization of the recombinant protein^82^. In addition, researchers have detected another O‐glycosylation site at Thr678, which is positioned next to the polybasic furin cleavage site between the S1 and S2 subunits^83^. Moreover, based on predictive analysis, it has been suggested that SARS‐CoV‐2 S contains five potential O‐glycosylation sites (Ser673, Thr678, Tyr28‐Arg34, Thr1160, and Ser686). These O‐glycosylation sites may play a role in shaping the MLD, potentially providing protection against S protein epitopes or acting as crucial residues for evading the immune system's response to SARS‐CoV‐2^84^, ^85^.

As part of ongoing efforts to address the challenges posed by the low stoichiometry of O‐glycosylation on SARS‐CoV‐2 S, researchers developed Trp‐Arg monomer‐grafted silica microspheres (referred to as WR‐SiO_2_) for analyzing the O‐glycosylation profile of SARS‐CoV‐2 S. This analysis led to the identification of 27 O‐glycosylation sites, including 18 sites that were unambiguously determined^86^. To address the challenges faced by conventional bottom‐up glycoproteomic approach, a biomimetic polymer with specific selectivity for O‐linked glycopeptides has been developed. This polymer effectively minimizes interference from nonglycopeptides during O‐linked glycopeptide enrichment. To address the limitations of conventional methods, a top‐down MS approach combining native and denaturing techniques has been utilized. This approach merges trapped ion mobility spectrometry quadrupole time‐of‐flight (TIMS‐QTOF) with ultrahigh‐resolution Fourier transform ion cyclotron resonance (FTICR)‐MS. This enables a thorough evaluation of glycoforms, facilitating the definitive identification of both glycan structures and their corresponding glycosites. For the first time, the structural elucidation of eight O‐glycoforms of the S GP, along with their relative molecular abundance, has been achieved. This breakthrough paves the way for understanding the functional roles of emerging SARS‐CoV‐2 S‐RBD variants and other O‐glycoproteins^86^. In addition to O‐glycoproteomic profiling, the novel dual‐functional immobilized titanium ion affinity chromatography (Ti‐IMAC) enrichment approach drew the similar conclusion above that Thr323 and Ser325 at the RBD could be potential O‐linked glycosylation sites^87^. By adopting this innovative approach, the interference caused by neutral glycopeptides can potentially be eliminated, leading to improved coverage of glycoforms in the S protein. It is important to highlight that these O‐linked glycosylation sites are situated in close proximity to two N‐linked glycosylation sites, namely Asn331 and Asn343. Additionally, researchers have accurately mapped the specific positions of O‐linked glycosylation sites on three distinct components of the S protein: ectodomains produced in insect cells, ectodomains produced in human cells, and RBD derived from insect cells. In total, 25 O‐glycosites were identified, with an intriguing observation that 16 out of these 25 O‐glycosites were found within a mere three amino acids from known N‐glycosites^88^. Intriguingly, Gao et al. also reported a similar phenomenon where N‐ and O‐linked glycosylation occurred together in positions associated with N‐sequons, leading to the proposal of an “O‐Follow‐N” rule^89^.

The precise functions of glycosylation in the densely glycosylated S protein of SARS‐CoV‐2 remain uncertain. First, it is speculated that glycosylation may regulate proteolysis of the S protein. Following the binding of the S protein's RBD region to ACE2, viral fusion and replication are facilitated by the furin/RRAR‐S site located between the S1 and S2 subunits of S protein^90^. Second, further research has revealed that O‐glycans can impact viral entry by influencing proteolysis rates at the S1–S2 interface and potentially inhibiting O‐glycan elaboration^91^. O‐glycans found on the S protein might partially contribute to the recognition of various innate immune receptors, such as macrophage galactose/GalNAc specific lectin (MGL) CLEC10A/CD301^84^. Although NA treatment only slightly enhanced the binding, it was observed that sialic acid seemed to cover or mask certain MGL ligands. This finding implies that O‐glycans may play a partial role in the recognition of NA. Third, it was observed by researchers that O‐glycosylation mediated by specific members of the GALNT family could inhibit the furin cleavage of the S protein and the formation of syncytia. This type of O‐glycosylation relies on Pro681. Their findings indicated that mutations at Pro681 would diminish O‐glycosylation, leading to increased furin cleavage and syncytia formation. This partly elucidates why the alpha and delta variants carrying the Pro681 mutants exhibited heightened transmissibility^92^.

MD simulations have also been proven valuable in O‐glycosylation studies. For instance, a novel mutant, Asp494Ser, on the RBD of the S protein, was introduced to generate O‐glycosylated Ser494. MD simulations revealed that Ser494 glycosylation led to a stabilized interaction between the RBD and ACE2, resulting in an enhanced binding affinity of the RBD to ACE2, which was dependent on the size of the attached O‐glycan^93^. Moreover, an atomistic model of the SARS‐CoV‐2 RBD–ferritin nanoparticle vaccine was developed, demonstrating that the addition of O‐glycans to the ferritin–RBD interface improved the stability of the ferritin‐RBD complex. This finding could be utilized to optimize the nanoparticle vaccine^94^. Given the challenges associated with the chemical synthesis of GPs, MD simulations, facilitated by tools like AlphaFold, offer a beneficial approach to understanding how glycans may influence protein–protein interactions.

New O‐glycosylation sites are being revealed on the SARS‐CoV‐2 variant, known as Omicron. A recently discovered O‐glycosite (Thr376) exclusive to the Omicron variant has been identified by researchers. Additionally, they directly measured the quantities of core 1 and core 2 O‐glycan structures and investigated the structural diversity of O‐glycoforms among the three variants^95^. Moreover, it has been demonstrated that the Asn679Lys mutation in the Omicron strain enhances the likelihood of O‐linked glycosylation at the S1/S2 cleavage site, potentially impeding viral entry through the cell surface. This, in turn, could reduce syncytia formation and promote cell entry through the endocytic pathway^96^.

In addition to the S protein, researchers have also discovered that ACE2 undergoes O‐glycosylation. The peptide that includes Thr730 in ACE2 is almost entirely taken up by core 1 mucin‐type O‐glycan GalNAcGalNeuAc2, which accounts for approximately 97% of its composition (Figure 3). Monosialylated GalNAcGalNeuAc glycans are also prevalent on ACE2^97^.

We speculate that there may be novel O‐glycosylation sites on SARS‐CoV‐2 S, ACE2, or within the intracellular trafficking system of host cells, which could play a role in viral intake and release. As researchers strive to map the complete viral glycan signature, they also aim to unravel the mechanisms underlying the occupancy and processing of O‐glycans. Recently, it has been discovered that the quaternary protein architecture may play a crucial role in limiting O‐glycosylation of the S protein in SARS‐CoV‐2^98^. For instance, it has been observed that a specific O‐linked glycan at Thr323 has a significantly lower occupancy in the native‐like trimer structure. However, when the RBD is produced as a monomer, multiple O‐linked glycosylation sites, including Thr323, show nearly complete glycan occupancy. This discrepancy in glycosylation patterns could be attributed to the steric restrictions imposed by the relaxed quaternary protein architecture^98^. Interestingly, their findings do not support the previously proposed “O‐Follow‐N” rule. The difference observed might be due to the incomplete removal of residual HexNAc remnants from N‐linked glycan degradation, which are not entirely eliminated by PNGase F, usually used to remove N‐linked glycans^1^. To provide a clear identification of O‐linked HexNAc in proximity to N‐glycan, it would be useful to simultaneously investigate N‐linked modifications and effectively fragment the N sequon. We aspire that our exploration of the world of glycans will enhance comprehension of the life cycle of virus and assist in the development of effective treatments.

It is crucial to acknowledge that both the S protein and ACE2 receptor of SARS‐CoV‐2 undergo significant O‐glycosylation. The O‐GlcNAc modifications on the S protein have been demonstrated to play a role in immune evasion and regulate proteolysis of the S protein, influencing viral entry. Furthermore, O‐glycans are crucial for binding with innate immune receptors. Reduction in O‐glycans can lead to suppressed viral spread through decreased furin cleavage of the S protein and reduced formation of syncytia. Additionally, O‐glycosylation stabilizes the interaction between RBD and ACE2, enhancing their binding affinity. Intriguingly, in the SARS‐CoV‐2 Omicron variant, O‐linked glycosylation has been found to hinder viral entry and reduce syncytia formation^96^. Further investigation into the functions of O‐GalNAc modifications involved in SARS‐CoV‐2 infection would be of great interest.

In summary, O‐glycosylation plays a significant role in the viral life cycle, including viral attachment and entry, assembly and release, spread within host cells, and modulation of the host's innate and adaptive immunity. Notably, O‐glycosylation has been shown to enhance viral replication in various viruses such as HBV, HSV, KSHV, HTLV‐1, IV, and RSV. In viruses such as HSV, EBOV, HCV, HTLV‐1, HeV, and SARS‐CoV‐2, it is also essential for viral attachment and entry. Furthermore, in the cases of HBV and HCV, O‐glycosylation modulates the viral assembly process. Additionally, O‐glycosylation is involved in the innate and adaptive immune responses induced by different viruses, including HSV, KSHV, EBOV, HIV‐1, IV, and SARS‐CoV‐2.

## EXPLOITING VIRAL O‐GLYCOSYLATION IN THERAPIES: FRIEND OR FOE

### Targeting O‐glycosylation in anti‐DNA virus therapies

Typically, IFNs form the primary defense against viruses. However, researchers recently found that at epithelial surfaces, there is an innate antiviral pathway induced by DNA virus infection before the IFNs. O‐glycans derived from DNA viruses have the ability to enhance the expression of chemokines associated with the C–X–C motif chemokine receptor 3 (CXCR3), thereby promoting antiviral activity that relies on neutrophils^99^. Thus, the O‐glycan–CXCR3 pathway might be unique to DNA viruses and could be exploited for therapeutics.

In the case of HBV, to develop prophylactic and therapeutic vaccines, researchers generated a preS virus‐like particle (VLP) to investigate the preS antigen. They identified that preS in VLP possessed two glycosylation sites, N‐glycosylation at Asn112 and O‐glycosylation at Ser98, which displayed significantly increased immunogenicity compared to recombinant preS, producing strong anti‐preS neutralizing antibodies^100^. Moreover, O‐glycosylation is also vital to the diagnosis of HBV infection. A quantitative immunoassay was devised by researchers to measure O‐glycosylated HBsAg, and they demonstrated that alterations in the levels of O‐glycosylated HBsAg in serum might indicate the cumulative levels of HBV DNA and RNA virions in the bloodstream^101^. Furthermore, O‐glycosylation of the PreS2 domain of M‐HBsAg could also be recognized as a distinct feature of genotype C HBV virions through a glycan‐based immunoassay^102^. Based on this, researchers developed a new Glyco‐PS2 antibody that recognized O‐glycosylated M‐HBsAg of genotype C to impede HBV infection^103^.

Targeting O‐glycosylation could be a strategy for treating HCC. Given that HCC is primarily linked to HBV and HCV, and IGF‐II is recognized as a serum marker for human HCC, it has been observed that O‐GlcNAc modification of IGFBP‐6 at Ser204, induced by HCV/HBV infection, reduces its binding with IGF‐II, leading to increased cellular expression of IGF‐II and the advancement of HCC. This discovery opens up the possibility of developing new therapies aimed at attenuating the risk of HCC by modulating the actions of IGF‐II during viral infections^104^.

O‐glycosylation is also vital for VZV vaccination. The envelope proteins of VZV are shown to be highly immunogenic, and gE is one of the most abundantly expressed proteins. Recently, researchers developed a recombinant subunit vaccine (Shingrix®), which showed higher protective efficacy by decreasing the number of interfering O‐glycans so that the remaining O‐glycan signature could enhance antibody binding^26^.

### EZH2 inhibitors in treating neurotropic viruses

A recent intriguing discovery has linked neurotropic viruses with O‐GlcNAcylation of an epigenetic regulator called enhancer of zest homolog 2 (EZH2). EZH2 is an enzyme responsible for histone methylation, specifically H3K27me2/3, which results in the suppression of gene transcription. Certain neurotropic viruses, such as rabies virus (RABV), vesicular stomatitis virus (VSV), Semliki Forest virus (SFV), and herpes simplex virus (HSV), possess the capability to invade the central nervous system (CNS). RABV and VSV are −RNA viruses, SFV is a +RNA virus, and HSV is a DNA virus. Through infecting Neuro‐2a (N2a) cells with RABV and utilizing deep RNA‐seq, researchers identified a virus‐induced long noncoding RNA (lncRNA) called EZH2 degradation associated lncRNA (EDAL)^105^. Mechanistically, EDAL binds to the vicinity of the mouse EZH2 Thr309 site, which is homologous to the human EZH2 Thr313 O‐GlcNAcylation site^106^. By blocking EZH2 Thr309 O‐GlcNAcylation, EDAL induces EZH2 degradation and reduces H3K27me3 levels. This alleviates the H3K27me3 mark on an antiviral gene called PCP4L1, ultimately leading to an antiviral response in the host^105^. This discovery holds potential implications for antiviral therapy. The EZH2 inhibitor Gsk126 has been shown to significantly suppress the replication of rRABV and VSV in N2a cells^105^. This finding suggests a promising new strategy for combating neurotropic viruses.

### O‐GlcNAc in anti‐RNA virus innate immunity

In the battle against RNA viral infections, host cells activate the antiviral immune response, and OGT plays distinct roles in host defense^107^. Cytosolic RNA is sensed by retinoic acid‐inducible gene I‐like receptors (RLRs), triggering the oligomerization and O‐GlcNAcylation of the adaptor protein mitochondrial antiviral‐signaling protein (MAVS). This O‐GlcNAcylation event is essential for MAVS Lys63‐ubiquitination and subsequent activation of the downstream RLR antiviral signaling pathway^108^, ^109^, ^110^.

In the context of VSV infection, there is an observed increase in total O‐GlcNAcylation^108^. Notably, MAVS is O‐GlcNAcylated at Ser366. Depletion of the enzyme OGT results in defective activation of the RLR‐mediated antiviral immune signaling and reduced production of inflammatory cytokines in response to VSV infection^108^. Furthermore, in the cases of IAV and VSV, depletion of O‐GlcNAc levels renders host myeloid cells more susceptible to viral infection. Notably, administration of d‐glucosamine, known to boost O‐GlcNAcylation, has demonstrated protective effects against viral infection in mouse models^110^.

In the case of Sendai virus (SeV) infection, cellular O‐GlcNAcylation levels decrease, and MAVS is found to be O‐GlcNAcylated between amino acid residues 249 and 257. This O‐GlcNAcylation event inhibits the activation of downstream events^109^. Therefore, based on the limited available reports^108^, ^109^, ^110^, O‐GlcNAcylation of MAVS at Ser366 promotes the host defense mechanism, at least during VSV and IAV infection. Conversely, O‐GlcNAcylation of MAVS between amino acid residues 249 and 257 appears to suppress the host defense mechanism during SeV invasion. It should be emphasized that there could be undiscovered O‐GlcNAcylation sites on MAVS. Additionally, the situation becomes complicated when multiple O‐GlcNAcylation sites on the same protein have varying biological functions. It is likely that more components involved in the RLR‐mediated viral infection pathway are O‐GlcNAcylated, but a meticulous examination is required to determine their individual modification sites and precise functions. Nonetheless, researchers have proposed a therapeutic approach that combines the use of azithromycin, which enhances RNA virus‐induced type 1 IFN response, with high‐dose glucosamine.

### A sweet war against HIV

There are also many strategies against HIV by targeting O‐glycosylation in therapies. Focusing on HIV gene expression, investigators found that the transcription factor Sp1 was O‐GlcNAcylated, which could bind to the region of HIV 5′ LTR. Overexpression of OGT or supplement of glucosamine to enhance O‐GlcNAcylation inhibited the HIV LTR promoter in immune cells, suggesting that OGT‐Sp1‐HIV LTR could regulate HIV latency and activation, which gave the potential to establish a metabolic supplement for HIV therapy^111^.

Moreover, it was discovered that the administration of benzyl‐2‐acetamido‐2‐deoxy‐α‐d‐galactopyranoside (BAGN), an inhibitor of O‐glycosylation commonly used in various cell lines, resulted in an elevated proportion of HIV‐infected cells, an increased quantity of HIV p24 protein per cell, and higher levels of viral particles in supernatants. This finding could potentially offer new perspectives on therapeutic targets related to O‐glycosylation for strategies aimed at controlling HIV^112^.

Furthermore, a comprehensive understanding of O‐glycosylation in HIV prompts researchers to develop better vaccines. One promising candidate for developing new vaccines is HIV‐1 VLP, which mimics the native structure of the virus without being infectious. To optimize the design of VLP‐based vaccines using glycoengineering methods, researchers examined the O‐glycosylation patterns of Gag VLPs produced in HEK293 cells^113^. They utilized a combination of porous graphitized carbon separation and MS analysis to identify different glycan profiles between VLPs and extracellular vesicles. This characterization provides valuable insights into the immunogenic potential of VLPs and paves the way for future development of VLP‐based vaccines^113^.

## CONCLUDING REMARKS

PTM through O‐glycosylation is a crucial process that significantly impacts the physiochemical properties of proteins, spanning from viruses to humans. Despite being less studied than N‐linked glycans, O‐linked glycans, which have revealed their involvement in various aspects of viral life cycle and humoral immune response (Table 1). Therefore, this review aims to summarize the literature on O‐glycosylation related to viruses occurring in viral and host proteins, as well as recent advancements in therapeutic GPs for antiviral purposes.

**Table 1:** O‐Glycosylation sites in viral proteins and their potential functions.

Virus	Viral proteins	O‐Glycosylation sites	Potential functions	References
EBOV	NP		Formation of viral particles.	[50]
EBV/BoHV‐4	gp350/gp180		Virus binding to B cells, a neutralization target.	[43]
HBV	preS	Ser98	Higher immunogenicity.	[100]
HeV	G		Trigger of fusion process, effect on G/F interactions, G conformation, receptor‐induced G conformational changes, F processing and F incorporation into pseudotyped virions.	[78]
HIV	Env/gp120/gp41	Thr499, Thr606	Evasion of neutralizing antibodies and the humoral immune response.	[60, 68]
HSV‐1	gB	Thr53, Thr480	PILRα‐dependent viral entry, enhancement of viral replication and virulence	[29]
	gC		Viral attachment to susceptible host cells, virus diffusion, accelerated virus penetration into cells, and modulation of selectin ligands expression.	[17, 24, 25]
	gD		Activating plasmacytoid dendritic cells.	[31, 37]
IAV	HA1	Thr12, Thr16	Virus replication, inflammation inhibition, and enhancement of HBP flux.	[62, 73]
KSHV	ORFs		Viral DNA synthesis, viral replication and propagation.	[39, 41]
SARS‐CoV‐2	Spike protein	Thr323, Ser325, Thr678, Ser673, Thr678, Tyr28‐Arg34, Thr1160, Ser686	Maintenance of RBD as a monomer, immune‐evasion, viral fusion and entry, transmission, suppressing the furin cleavage of S and the formation of syncytia.	[81, 82, 83, 84, 87, 98]

The glycan structures can be exploited for therapeutic purposes^114^. However, great challenges remain due to the innate complexity of glycans, such as inherent weak binding between glycans and proteins, heterogeneous glycoforms of various glycan structures, and the difficulty in telling apart viral glycans from the “self” host glycans^115^. Nevertheless, progress has been made to utilize carbohydrate‐binding reagents or synthetic carbohydrate ligands for treating viral diseases. A case in point is using high‐dose mannose‐binding lectins (targeting N‐glycans) to boost the survival of EBOV‐infected mice^116^.

O‐glycans are even more difficult to fully understand than N‐glycans, as they have no consensus motif, which renders identification of the modified sites heavily relying on MS^117^. O‐glycosylation studies in viruses have just begun, and already an OGT inhibitor has been demonstrated to dampen the yields of herpesviruses^33^, indicating its great potential for further clinical utilizations. Admittedly, the field is still burgeoning with many questions unanswered: What are the O‐glycosylation sites on the various virions? Do the different O‐glycosylation modifications share a common theme of molecular mechanisms? Can we develop specific drugs, and if so, what is their efficacy? In recent times, MS has emerged as a powerful and widely used tool in combination with several techniques to characterize protein glycosylation. Insights into glycomics can be gained by utilizing various methods alone or in combination. One such approach is to investigate noncovalent interactions between specific proteins and glycans. This can be achieved by selectively identifying glycan epitopes or motifs in tissues through the use of antibodies or lectins, enabling an assessment of the functional roles of glycans. Furthermore, structural information about glycans can also be obtained through a glycomic workflow. This involves releasing glycans from GPs using chemical or enzymatic techniques to reveal their structural details^118^. However, it is important to acknowledge the limitations associated with these glycomic approaches. One limitation is the potential loss of protein‐ and site‐specificity upon glycan release.

In the future, improved tools for O‐glycosylation detection, more precise MD simulations, and virtual screening techniques may enable the development of tailored antiviral therapeutics targeting O‐glycans. Concurrently, the significance of O‐glycosylation in oncogenic viruses is also gaining attention. The findings from this research have the potential to enhance our comprehension of tumor pathology, expand the repertoire of biomarkers available for diagnosis, and identify valuable molecular targets for effective therapy.

While O‐glycosylation modifications have been well characterized in some viruses, they are often overlooked or considered as a component of the complex interactions and evolutionary dynamics between hosts and viruses. Nevertheless, as zoonotic viruses continue to emerge and pose significant risks to public health, there is a growing need to pinpoint the factors that influence biological limitations in cross‐species transmission. In this context, the comparative zoology of glycosylation has started to be explored, although the involvement of O‐glycosylation in this process and its relationship to zoonotic risk are not yet fully elucidated. Incorporating more glycomics into viral surveillance could be a viable approach to establish a foundation for epidemic preparedness, particularly at the animal–human interface.

In summary, viruses utilize glycosylation as a means to survive and propagate. It is our hope that by targeting this intricate pathway, we can alleviate symptoms and develop effective medical interventions to combat viral infections.
